# Association Between Early Weight Loss and One-Year Weight Regain After Lifestyle-Based Obesity Treatment: A Retrospective Cohort Study

**DOI:** 10.3390/medicina62081450

**Published:** 2026-07-26

**Authors:** İbrahim Doğan, Serpil Sevimli Deniz, Mehmet Kadir Bartın, Ezgi Sönmez, Meltem Ceylan Delice, Serdar Çoban, Sebahattin Çelik

**Affiliations:** 1 Department of General Surgery, Van Training & Research Hospital, University of Health Sciences, 65170 Van, Türkiye; mehmetkadir.bartin@sbu.edu.tr (M.K.B.); ezgi.sonmez1@sbu.edu.tr (E.S.); sebahattin.celik@sbu.edu.tr (S.Ç.); 2 Department of Statistics, Institute of Science, Van Yuzuncu Yil University, 65080 Van, Türkiye; sdeniz@yyu.edu.tr; 3 Department of Anesthesiology and Resuscitation, Van Training & Research Hospital, University of Health Sciences, 65170 Van, Türkiye; meltem.ceylan@sbu.edu.tr; 4 Department of Surgical Oncology, Van Training & Research Hospital, University of Health Sciences, 65170 Van, Türkiye; serdar.coban@saglik.gov.tr

**Keywords:** obesity, early weight loss, weight regain, lifestyle intervention, weight maintenance, retrospective cohort

## Abstract

*Background and Objectives:* Sustained weight loss remains one of the greatest challenges in obesity management, as many individuals experience weight regain after initial treatment success. Although early weight loss has consistently been associated with greater overall weight reduction, its relationship with subsequent weight regain in routine clinical practice remains insufficiently characterized. This study examined the association between early weight loss and clinically significant weight regain among adults participating in a lifestyle-based obesity management program. *Materials and Methods:* We conducted a retrospective cohort study of adults receiving structured lifestyle-based obesity treatment at a tertiary care obesity clinic. Early weight loss was defined as the percentage reduction in body weight from baseline to the routinely scheduled third-month follow-up measurement. Clinically significant weight regain at one year was defined as regaining at least 50% of the maximum weight lost from baseline to nadir weight, expressed as the weight regain ratio (WRR ≥ 0.50). Logistic regression was used to examine the association between early weight loss and subsequent weight regain after adjustment for age, baseline body mass index, and baseline body weight. Sensitivity analyses included a female-only model, an alternative weight regain definition (WRR ≥ 0.25), and Firth-penalized logistic regression. *Results:* Among 79 participants with complete follow-up data, 9 (11.4%) experienced clinically significant weight regain at one year. *Conclusions:* Greater early weight loss was independently associated with lower odds of clinically significant weight regain (adjusted odds ratio 0.784, 95% confidence interval 0.646–0.950; *p* = 0.013). The association remained consistent in the female-only analysis and in the Firth-penalized logistic regression. When clinically significant weight regain was alternatively defined as WRR ≥ 0.25, the association remained in the same direction but did not reach statistical significance. Age, baseline body mass index, and baseline body weight were not significantly associated with the outcome.

## 1. Introduction

Obesity is a chronic, progressive, and relapsing disease characterized by excessive or abnormal adiposity that adversely affects health and is associated with increased morbidity, mortality, and healthcare costs worldwide [1,2,3,4,5,6,7]. According to the World Health Organization (WHO), obesity has reached epidemic proportions, with nearly one billion adults living with obesity globally, making it one of the most pressing public health challenges of the twenty-first century [3]. Beyond its well-established association with type 2 diabetes mellitus, cardiovascular disease, metabolic dysfunction-associated steatotic liver disease, obstructive sleep apnea, several malignancies, and reduced quality of life, obesity is increasingly recognized as a chronic disease requiring lifelong management rather than a condition that can be successfully treated through short-term weight reduction alone [1,2,3,4,5,6,7].

Lifestyle modification remains the cornerstone of obesity management and is recommended as first-line therapy by all major international clinical practice guidelines [1,2,4,6,7,8]. Comprehensive lifestyle interventions, integrating individualized dietary counseling, increased physical activity, behavioral modification, and regular clinical follow-up, consistently achieve clinically meaningful weight loss and improve cardiometabolic health [1,6,9]. Nevertheless, despite initial treatment success, maintaining weight loss over time remains a major therapeutic challenge. Long-term follow-up studies have demonstrated that many individuals regain a substantial proportion of the weight initially lost, resulting in attenuation of metabolic improvements and increased risk of disease recurrence [9,10,11,12,13,14,15,16,17,18,19]. Consequently, preventing weight regain has become a central objective of long-term obesity management.

The biological mechanisms responsible for weight regain are complex and multifactorial. Following weight loss, the human body activates a series of adaptive physiological responses that promote restoration of lost weight. These responses include reductions in resting energy expenditure, increased metabolic efficiency, persistent alterations in appetite-regulating hormones, enhanced hunger, reduced satiety, and increased reward-driven eating behavior [11,12,13,20,21,22,23]. Importantly, these neuroendocrine adaptations may persist for several years after weight reduction, creating a biological environment that favors weight regain despite continued behavioral efforts [13]. However, biological mechanisms alone do not fully explain long-term weight trajectories. Behavioral adherence, psychological factors, physical activity patterns, socioeconomic conditions, environmental influences, and access to continuous clinical support all contribute substantially to the considerable interindividual variability observed after obesity treatment [1,5,10,24]. This complexity has shifted the contemporary understanding of obesity from a simple energy imbalance to a chronic relapsing disease requiring individualized, long-term management strategies.

Because long-term weight maintenance remains difficult, considerable attention has focused on identifying early clinical markers associated with subsequent treatment outcomes. Among these, early weight loss achieved during the first weeks or months of treatment has consistently emerged as one of the strongest indicators of overall treatment success [14,15,16,25,26]. Previous randomized controlled trials and behavioral intervention studies have shown that individuals who achieve greater early weight loss generally experience greater total weight reduction and improved long-term weight maintenance [14,15,16,25,26]. Consequently, early treatment response has been proposed as a pragmatic clinical indicator that may help clinicians identify patients who require more intensive follow-up or additional therapeutic interventions.

Despite this evidence, relatively few studies have specifically investigated the relationship between early weight loss and clinically significant weight regain as a distinct clinical outcome. Weight-loss success and weight regain represent related but conceptually different phases of obesity management. Whereas initial weight loss primarily reflects treatment responsiveness, weight regain reflects the long-term interaction between adaptive biological responses, behavioral adherence, and environmental influences that emerge after initial weight reduction [12,13,18,20]. Therefore, determinants of successful weight loss may not necessarily be identical to determinants of subsequent weight regain.

Interpretation of the existing literature is further complicated by substantial methodological heterogeneity. Published studies have used different definitions of clinically significant weight regain, various reference body weights (baseline weight, nadir weight, or maximum weight loss), heterogeneous follow-up periods, and diverse analytical approaches [18,27]. Recognizing this limitation, the International Federation for the Surgery of Obesity and Metabolic Disorders (IFSO) recently emphasized the importance of standardized definitions and reporting standards for obesity outcomes to improve comparability across studies and facilitate interpretation of long-term treatment results [2]. Furthermore, much of the available evidence originates from randomized clinical trials or highly controlled intervention programs, whereas considerably less evidence has been generated from routine clinical practice, where patient adherence, treatment intensity, and follow-up attendance are inherently more variable [9,10].

Another important consideration is the marked heterogeneity of treatment response among individuals with obesity. Patients presenting with similar demographic characteristics and baseline body mass index frequently exhibit markedly different long-term weight trajectories despite receiving comparable lifestyle interventions. This variability likely reflects complex interactions among genetic susceptibility, adaptive metabolic responses, behavioral adherence, psychological characteristics, environmental exposures, and social determinants of health [1,10,11,12,13,24,28]. Consequently, early weight loss should not be interpreted as a deterministic predictor of future outcomes but rather as one component of a multifactorial process influencing long-term body weight regulation.

Real-world observational studies provide complementary evidence to randomized trials because they reflect routine clinical practice, including variations in treatment adherence, multidisciplinary care, and follow-up attendance. Such studies may therefore improve understanding of weight trajectories in everyday obesity management and help identify patients who may benefit from individualized follow-up strategies. Nevertheless, observational studies also require cautious interpretation because selection bias, incomplete follow-up, and residual confounding may influence observed associations [29].

Therefore, the present study aimed to evaluate the association between early weight loss during the first three months of treatment and clinically significant weight regain at one year among adults participating in a structured lifestyle-based obesity management program in a tertiary-care obesity clinic. We hypothesized that greater early weight loss would be associated with a lower likelihood of clinically significant weight regain. Given the retrospective design, complete-case analysis, limited number of outcome events, and substantial loss to follow-up, the findings should be interpreted as exploratory and hypothesis-generating and should be confirmed in adequately powered prospective studies.

## 2. Materials and Methods

### 2.1. Study Design and Setting

This retrospective observational cohort study was conducted at the Obesity Center of Van Training and Research Hospital, University of Health Sciences Türkiye, Van, Türkiye, a tertiary referral center providing multidisciplinary obesity care in eastern Türkiye. The Obesity Center, established in 2022, delivers comprehensive evidence-based obesity management through a multidisciplinary team comprising endocrinologists, dietitians, physiotherapists, psychologists, and specialized nurses. Patients are managed according to current national and international obesity management recommendations, with individualized treatment plans based on clinical characteristics and patient needs.

The study was based on routinely collected clinical data from adults attending the Obesity Center after its establishment. The primary objective was to investigate the association between early weight loss achieved during the first three months of treatment and clinically significant weight regain after one year of follow-up under real-world clinical conditions.

The study was designed and reported in accordance with the Strengthening the Reporting of Observational Studies in Epidemiology (STROBE) Statement.

### 2.2. Study Population

Patients were eligible if they were 18 years of age or older, initiated the lifestyle-based obesity management program at the center, and had sufficient longitudinal anthropometric data to determine baseline body weight, body weight at the routinely scheduled third-month follow-up, nadir body weight during the first year of follow-up, and body weight at the routinely scheduled first-year follow-up.

Patients were excluded if they had undergone bariatric surgery before or during follow-up, used anti-obesity medications at baseline or at any time during the follow-up period, became pregnant during the observation period, had active malignancy, untreated hyperthyroidism, or any other medical condition known to substantially influence body weight. Individuals with incomplete clinical records or insufficient longitudinal weight measurements preventing calculation of the weight regain ratio (WRR) were also excluded.

**Figure 1:**
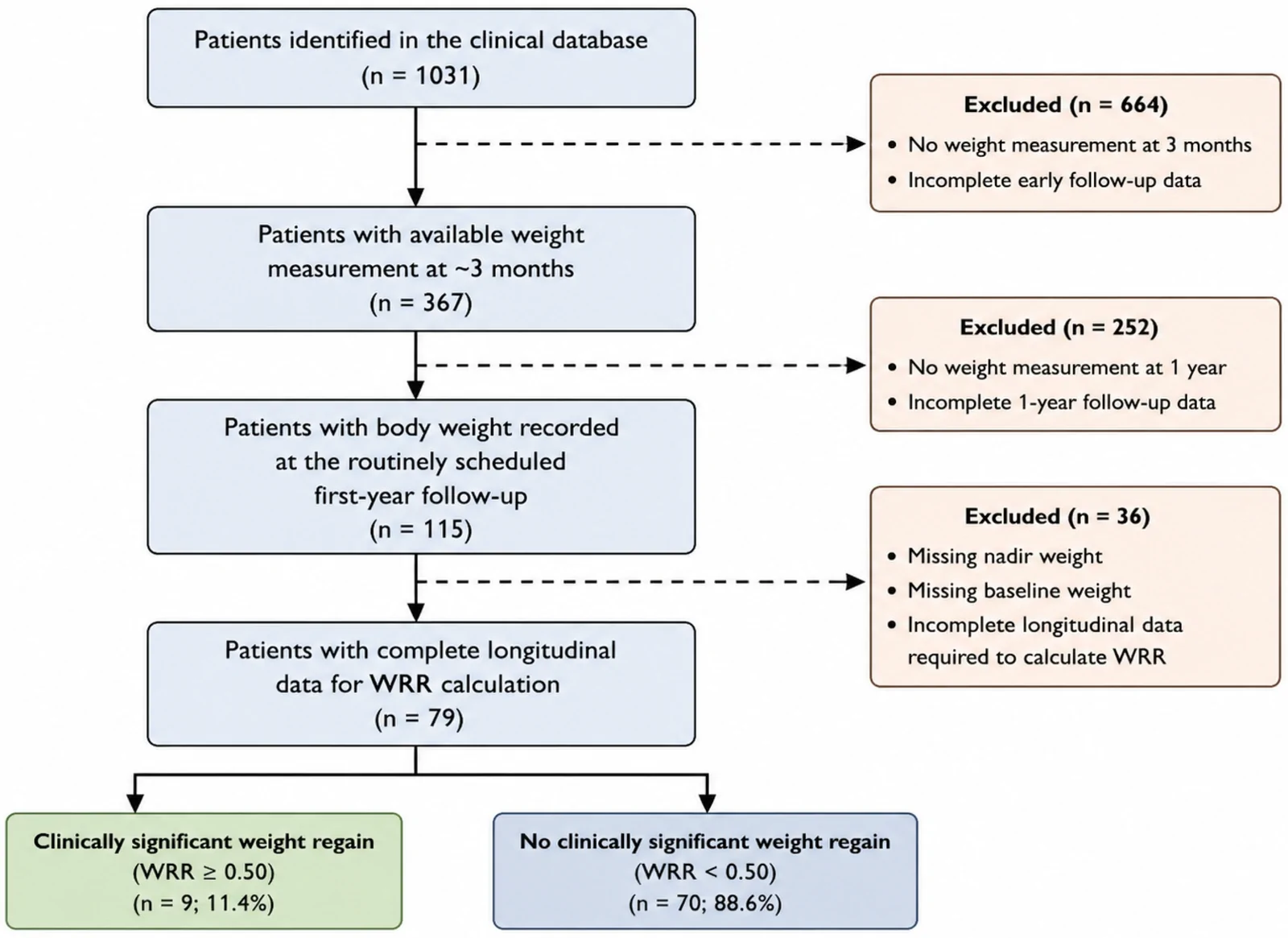
Flowchart of participant selection.

A total of 1031 adults with obesity who attended the Obesity Center were identified in the institutional clinical database. Of these, 367 had body weight measurements available at the routinely scheduled third-month follow-up, whereas 115 had body weight measurements available at the routinely scheduled first-year follow-up. Complete longitudinal data required to calculate the weight regain ratio (WRR) were available for 79 participants, who comprised the final analytical cohort (Figure 1).

Patients were routinely scheduled for monthly follow-up visits as part of the obesity management program. However, attendance reflected routine clinical practice, and therefore not all participants attended every scheduled visit. Consequently, only patients with complete longitudinal weight measurements from baseline through the first-year follow-up were eligible for the primary analysis. The analytical cohort therefore represents a subset of the original study population who remained engaged in routine clinical follow-up. To assess the potential for selection bias, baseline characteristics of included and excluded participants were compared.

The flow diagram illustrates the selection process of the study population and the derivation of the final analytical cohort included in the statistical analyses.

### 2.3. Lifestyle-Based Obesity Treatment Program

All participants received a structured multidisciplinary lifestyle intervention delivered as part of routine clinical care.

Nutritional management consisted of individualized dietary counseling provided by registered dietitians with the objective of achieving gradual and sustainable weight reduction while maintaining nutritional adequacy. Dietary prescriptions were individualized according to baseline body weight, metabolic status, eating habits, and personal preferences. Counseling emphasized caloric restriction, portion control, increased consumption of vegetables, fruits, whole grains, legumes, and lean protein sources, while reducing the intake of energy-dense ultra-processed foods and sugar-sweetened beverages.

Participants were encouraged to perform at least 150 min of moderate-intensity aerobic physical activity per week, supplemented with resistance exercises whenever clinically appropriate. Physical activity recommendations were individualized according to age, functional capacity, obesity severity, and accompanying medical conditions.

Behavioral support included education regarding self-monitoring of dietary intake and body weight, goal setting, stimulus control, problem-solving strategies, relapse prevention, and motivational counseling designed to improve long-term adherence to lifestyle modification.

Patients were routinely evaluated during outpatient follow-up visits, during which dietary adherence, physical activity habits, anthropometric measurements, and treatment response were reviewed. Patients were routinely scheduled for monthly outpatient follow-up visits. However, because the study reflects routine clinical practice, actual attendance varied according to individual adherence and clinical circumstances, resulting in differences in the timing and number of completed follow-up visits.

The treatment program evaluated in the present study consisted exclusively of non-pharmacological lifestyle modification, including individualized nutritional counseling, physical activity recommendations, and behavioral support. Patients who used anti-obesity medications at baseline or at any time during follow-up were excluded from the study.

### 2.4. Anthropometric Measurements

Anthropometric measurements were performed during routine outpatient visits by trained healthcare professionals following standardized clinical procedures.

Body weight was measured using calibrated digital scales with participants wearing light indoor clothing and no shoes. Height was measured at the initial visit using a wall-mounted stadiometer, and body mass index (BMI) was calculated as weight in kilograms divided by height in meters squared (kg/m^2^).

Baseline clinical variables extracted from the electronic medical records included age, sex, body weight, BMI, waist circumference, physical activity score, exercise frequency score, walking activity score, screen time score, glycated hemoglobin (HbA1c), fasting insulin concentration, and other routinely collected clinical and metabolic variables. Because several of these variables contained substantial missing data, only prespecified variables with adequate completeness were included in the primary statistical analyses.

### 2.5. Outcome Definition

Early weight loss was defined as the percentage reduction in body weight from baseline to the routinely scheduled third-month follow-up measurement. Because the study was based on routine clinical practice, attendance at scheduled follow-up visits varied among patients. Accordingly, only participants with an available third-month body weight measurement were eligible for assessment of early weight loss.

The nadir weight was defined as the lowest documented body weight recorded during the first year of follow-up.

The primary outcome was clinically significant weight regain assessed using the body weight recorded at the routinely scheduled first-year follow-up. Weight regain was quantified using the weight regain ratio (WRR) according to the following equation:

WRR = (Weight at one year − Nadir weight)/(Baseline weight − Nadir weight)

A WRR of 0.50 or greater, corresponding to recovery of at least 50% of the maximum weight lost, was considered clinically significant in accordance with previously published definitions. Values greater than one indicated that body weight exceeded the baseline value after reaching the nadir and were retained in the analyses because they represent substantial weight regain.

As a sensitivity analysis, the primary outcome was recalculated using an alternative threshold of WRR ≥ 0.25.

### 2.6. Statistical Analysis

Statistical analyses were performed using Python (version 3.11) within the Google Colaboratory (Google Colab) cloud computing environment. Data management and statistical analyses were conducted using the pandas, NumPy, SciPy, statsmodels, and scikit-learn libraries.

Continuous variables were evaluated for normality using the Shapiro–Wilk test together with visual inspection of histograms and quantile–quantile plots. Normally distributed variables are presented as mean ± standard deviation (SD), whereas non-normally distributed variables are reported as median and interquartile range (IQR). Categorical variables are presented as frequencies and percentages.

Baseline characteristics were compared between participants with and without clinically significant weight regain using the independent-samples *t*-test or the Mann–Whitney U test for continuous variables and Pearson’s chi-square test or Fisher’s exact test for categorical variables, as appropriate.

The association between early weight loss and clinically significant weight regain was evaluated using logistic regression analysis. Univariable logistic regression was initially performed to examine the crude association between early weight loss and the primary outcome. Subsequently, a multivariable logistic regression model was constructed including early weight loss as the primary independent variable and age, baseline BMI, and baseline body weight as prespecified covariates selected on the basis of clinical relevance and previous literature. Sex was not included in the adjusted model because no outcome events occurred among male participants, resulting in complete separation.

Adjusted odds ratios (Ors) with corresponding 95% confidence intervals (Cis) were reported. Given the limited number of outcome events, model complexity was intentionally restricted to minimize overfitting [30]. To further evaluate the robustness of the findings, Firth-penalized logistic regression was performed as a sensitivity analysis to reduce potential small-sample bias associated with sparse-event data [31]. Additional sensitivity analyses included a female-only analysis and repetition of the primary analysis using the alternative definition of clinically significant weight regain (WRR ≥ 0.25).

All statistical tests were two-sided, and a *p*-value < 0.05 was considered statistically significant.

### 2.7. Missing Data

Because calculation of the weight regain ratio required longitudinal body weight measurements at baseline, the routinely scheduled third-month follow-up, nadir weight during the first year of follow-up, and the routinely scheduled first-year follow-up, only participants with complete measurements at these predefined time points were eligible for the primary analysis. Consequently, a complete-case analysis was performed, and no statistical imputation of missing values was undertaken.

The substantial reduction in sample size from the source population to the final analytical cohort reflects the availability of complete longitudinal follow-up within routine clinical practice rather than protocol-driven research visits. Accordingly, the analytical cohort may represent individuals with greater adherence to scheduled follow-up, and the possibility of selection bias should be considered when interpreting the study findings.

### 2.8. Ethical Considerations

The study was conducted in accordance with the ethical principles of the Declaration of Helsinki and was approved by the Non-Interventional Clinical Research Ethics Committee of Van Training and Research Hospital, University of Health Sciences Türkiye (Approval No. GOKAEK/2025-10-16; approved on 19 December 2025).

Because this was a retrospective observational study based exclusively on anonymized routinely collected clinical data, the requirement for informed consent was waived by the Ethics Committee.

## 3. Results

### 3.1. Participant Selection

A total of 1031 adults with obesity who attended the Obesity Center were identified in the institutional clinical database. Of these, 367 had body weight measurements available approximately three months after treatment initiation, whereas 115 had body weight measurements available at approximately one year. Complete longitudinal data required to calculate the weight regain ratio (WRR) were available for 79 participants, who comprised the final analytical cohort (Figure 1).

Among the included participants, 9 (11.4%) experienced clinically significant weight regain during the first year of follow-up (WRR ≥ 0.50), whereas 70 (88.6%) did not.

To evaluate potential selection bias, baseline characteristics of included and excluded patients were compared (Supplementary Table S1). Compared with excluded patients, participants included in the analytical cohort were younger (38.1 vs. 41.0 years, *p* = 0.025) and had a higher baseline BMI (41.0 vs. 38.9 kg/m^2^, *p* = 0.002). Baseline body weight did not differ significantly between groups (*p* = 0.299). Although women were more frequently represented in the analytical cohort (96.2% vs. 88.9%), this difference was not statistically significant (*p* = 0.065). These findings suggest that the final cohort may represent individuals with greater adherence to follow-up within routine clinical practice.

### 3.2. Baseline Characteristics

Baseline demographic and anthropometric characteristics according to clinically significant weight regain status are summarized in Table 1.

Data are presented as mean ± standard deviation (SD) or number (percentage). Continuous variables were compared using the independent-samples *t*-test, and categorical variables were compared using Fisher’s exact test, as appropriate. Clinically significant weight regain was defined as a weight regain ratio (WRR) ≥ 0.50.

Patients who experienced clinically significant weight regain demonstrated substantially lower early weight loss than those who maintained their weight loss (2.4 ± 2.3% vs. 7.7 ± 5.5%, *p* < 0.001). In contrast, no statistically significant differences were observed between groups regarding age (39.3 ± 10.0 vs. 38.0 ± 11.0 years, *p* = 0.745), baseline BMI (38.7 ± 4.3 vs. 41.3 ± 5.4 kg/m^2^, *p* = 0.156), baseline body weight (96.3 ± 13.4 vs. 105.3 ± 15.0 kg, *p* = 0.110), or sex distribution (*p* = 1.000). As expected, the mean WRR was markedly higher among participants with clinically significant weight regain than among those without regain (1.02 ± 0.55 vs. 0.07 ± 0.12, *p* = 0.002).

**Figure 2:**
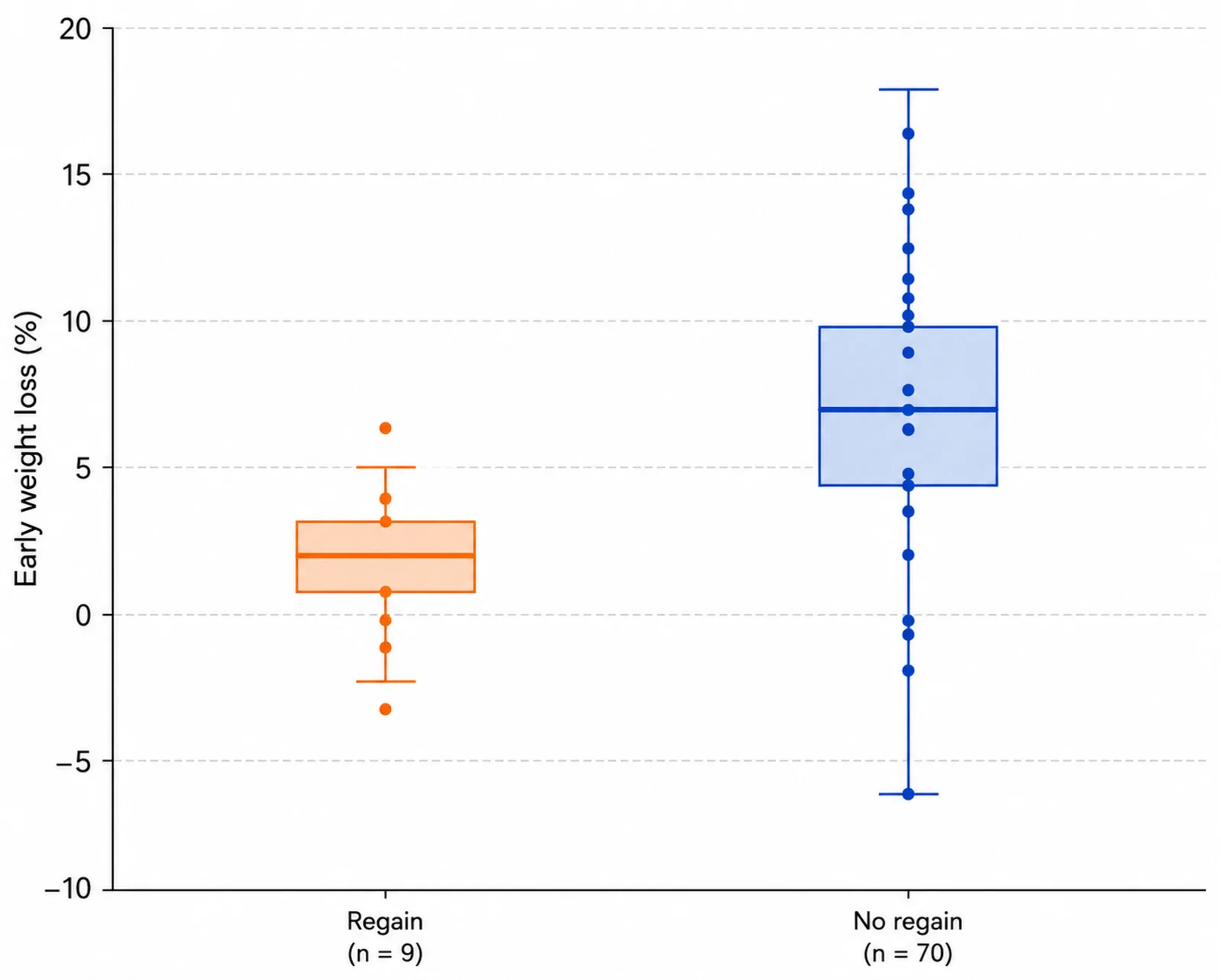
Distribution of early weight loss according to one-year weight regain status.

The distribution of early weight loss according to weight regain status is illustrated in Figure 2, demonstrating considerably lower early weight loss among participants who subsequently experienced clinically significant weight regain.

Participants who experienced clinically significant weight regain exhibited substantially lower early weight loss than those who maintained weight loss.

### 3.3. Weight-Loss Trajectory

The mean time to nadir body weight was 9.7 months (range, 3–12 months), indicating that the lowest body weight was generally achieved well after the routinely scheduled third-month follow-up. The mean maximum percentage weight loss achieved during follow-up was 23.5% ± 14.0% relative to baseline body weight.

### 3.4. Association Between Early Weight Loss and Clinically Significant Weight Regain

In the univariable logistic regression analysis, greater early weight loss was significantly associated with a lower likelihood of clinically significant weight regain after one year (Table 2).

Clinically significant weight regain was defined as a weight regain ratio (WRR) ≥ 0.50. Odds ratios are expressed per 1% increase in early weight loss.

Specifically, each 1% increase in early weight loss was associated with an 18.5% reduction in the odds of clinically significant weight regain (OR 0.815, 95% CI 0.688–0.965; *p* = 0.018).

After adjustment for age, baseline BMI, and baseline body weight, early weight loss remained independently associated with the primary outcome (adjusted OR 0.784, 95% CI 0.646–0.950; *p* = 0.013). None of the covariates included in the multivariable model showed statistically significant associations with clinically significant weight regain. Age (OR 1.036, 95% CI 0.957–1.120; *p* = 0.381), baseline BMI (OR 1.047, 95% CI 0.802–1.368; *p* = 0.734), and baseline body weight (OR 0.955, 95% CI 0.869–1.048; *p* = 0.332) were not independently associated with the outcome (Table 3).

Odds ratios are adjusted for age, baseline BMI, and baseline body weight. Clinically significant weight regain was defined as a weight regain ratio (WRR) ≥ 0.50. Odds ratios for early weight loss represent the change in odds associated with each 1% increase in early weight loss.

**Figure 3:**
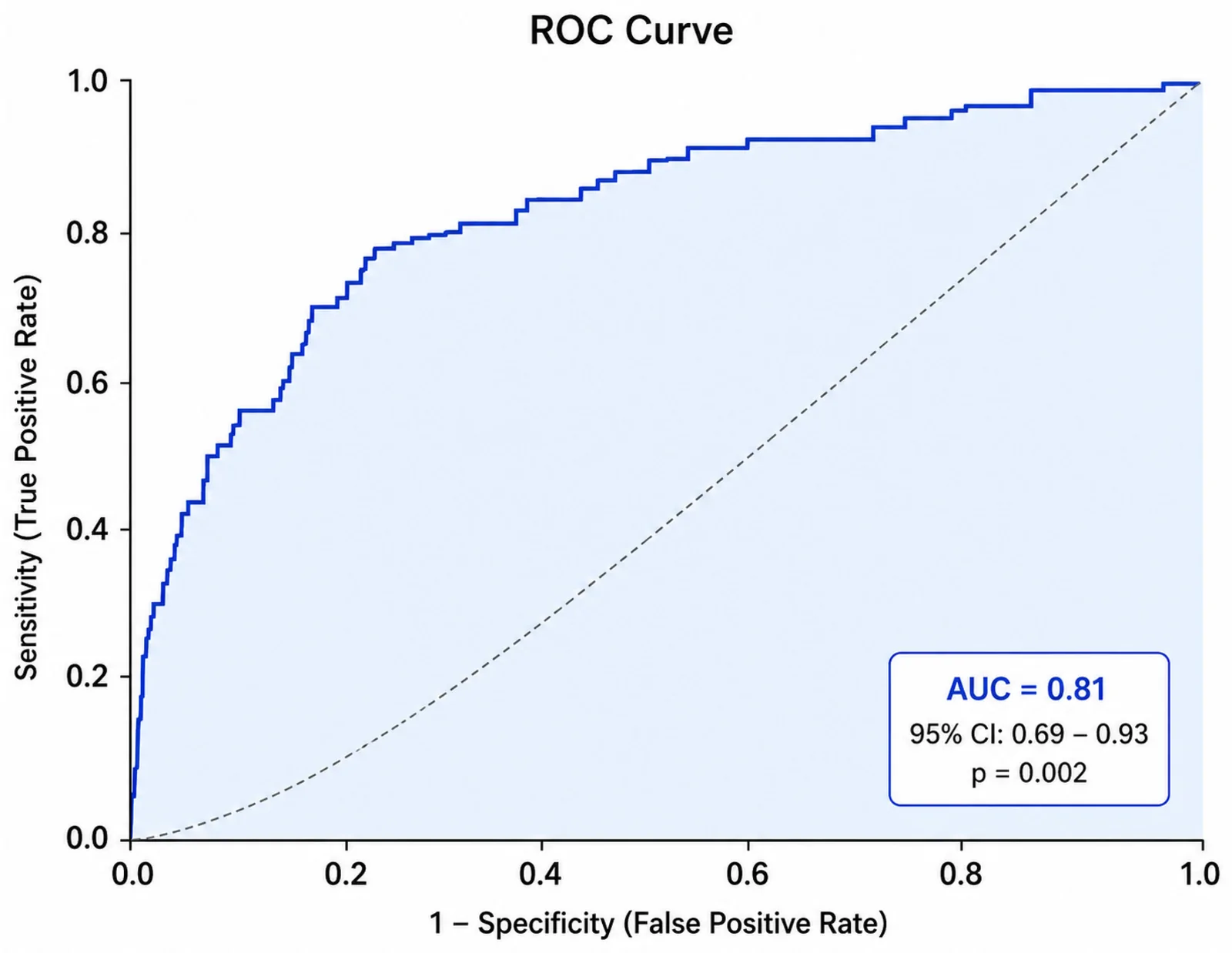
ROC curve showing the discriminatory ability of early weight loss for predicting clinically significant weight regain.

The apparent discrimination of the multivariable logistic regression model was good (AUC 0.81, 95% CI 0.69–0.92) (Figure 3). Because this performance was estimated in the development dataset, it should be interpreted as apparent model performance.

**Figure 4:**
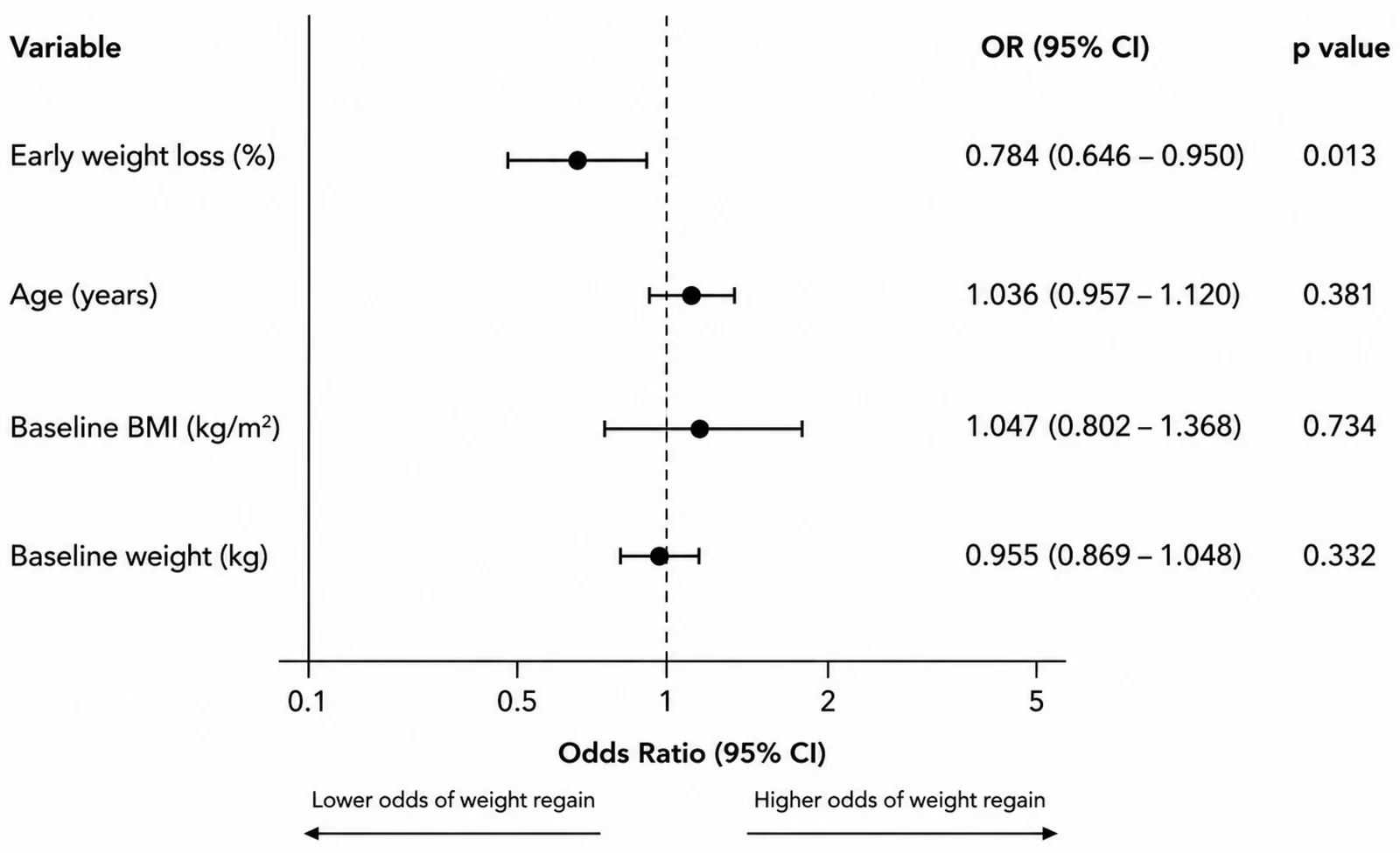
Forest plot of the multivariable logistic regression model (ORs with 95% confidence intervals).

None of the adjustment variables showed statistically significant associations with clinically significant weight regain (Table 3). The adjusted odds ratios and corresponding 95% confidence intervals from the multivariable logistic regression model are presented in Figure 4.

Adjusted odds ratios (ORs) and 95% confidence intervals (CIs) for predictors of clinically significant weight regain after one year.

### 3.5. Sensitivity Analyses

Sensitivity analyses generally supported the primary findings. Restricting the analysis to female participants and Firth-penalized logistic regression yielded results consistent with the primary analysis. When the outcome was redefined using WRR ≥ 0.25, the direction of the association remained similar; however, the association was attenuated and did not reach statistical significance. Restricting the analysis to female participants yielded similar results, with greater early weight loss remaining independently associated with a lower likelihood of clinically significant weight regain (OR 0.79, 95% CI 0.66–0.94; *p* = 0.008).

When clinically significant weight regain was redefined using a lower threshold (WRR ≥ 0.25), the direction of the association remained unchanged; however, the effect estimate was attenuated and no longer reached statistical significance (OR 0.95, 95% CI 0.87–1.05; *p* = 0.315) (Table 4).

The primary multivariable model was adjusted for age, baseline body mass index (BMI), and baseline body weight. Sensitivity analyses included restriction to female participants, application of an alternative definition of clinically significant weight regain (WRR ≥ 0.25), and Firth-penalized logistic regression to reduce potential small-sample bias. Odds ratios represent the change in the odds of clinically significant weight regain associated with each 1% increase in early weight loss.

Given the limited number of outcome events, a Firth-penalized logistic regression analysis was performed to assess the stability of the regression estimates. The results were highly consistent with those obtained using conventional logistic regression. Early weight loss remained independently associated with a lower likelihood of clinically significant weight regain (OR 0.814, 95% CI 0.686–0.966; *p* = 0.019), whereas age, baseline BMI, and baseline body weight remained non-significant predictors (Table 5).

Firth-penalized logistic regression was performed as a sensitivity analysis to reduce potential bias associated with sparse outcome events and small-sample logistic regression. The model included early weight loss, age, baseline body mass index, and baseline body weight. Odds ratios represent the change in the odds of clinically significant weight regain associated with each 1% increase in early weight loss.

## 4. Discussion

The present retrospective cohort study investigated the association between early weight loss during the first three months of a structured lifestyle-based obesity treatment program and clinically significant weight regain after one year in adults receiving multidisciplinary obesity care. The principal finding was that greater early weight loss was independently associated with a substantially lower likelihood of clinically significant weight regain, even after adjustment for age, baseline BMI, and baseline body weight. The primary findings were supported by the female-only analysis and the Firth-penalized logistic regression. Although redefining the outcome using WRR ≥ 0.25 yielded a similar directional association, statistical significance was not maintained. Taken together, these results suggest that early treatment response may provide clinically relevant prognostic information regarding subsequent weight maintenance in routine obesity care.

Early weight loss has long been recognized as one of the strongest predictors of overall treatment success in behavioral weight-management programs. Several randomized trials and longitudinal cohort studies have demonstrated that individuals achieving greater weight reduction during the initial weeks or months of treatment are more likely to achieve clinically meaningful long-term weight loss than those with a poor initial response [14,15,16,25,26]. However, relatively few investigations have specifically examined the relationship between early treatment response and subsequent weight regain as a distinct clinical outcome. Our findings therefore extend previous observations by demonstrating that early weight loss is associated not only with greater total weight reduction but also with a lower probability of regaining a substantial proportion of the lost weight during the first year of follow-up.

The magnitude of the observed association is clinically relevant. In the adjusted model, each 1% increase in early weight loss was associated with approximately a 22% reduction in the odds of clinically significant weight regain. Although the confidence intervals were relatively wide because of the limited number of outcome events, the consistency of the findings in the primary analysis, the female-only analysis, and the Firth-penalized logistic regression suggests that the observed association is unlikely to be explained solely by statistical instability. Furthermore, the apparent discrimination of the multivariable model (AUC 0.81) should be interpreted cautiously because it was estimated in the development dataset without external validation and may therefore overestimate predictive performance. Nevertheless, these findings support the potential value of early weight loss as a pragmatic marker for individualized follow-up strategies.

Several biological mechanisms may explain the observed relationship. Weight loss induces a coordinated series of neuroendocrine and metabolic adaptations that promote restoration of body weight. These adaptations include reductions in resting energy expenditure, increased skeletal muscle efficiency, alterations in circulating leptin, ghrelin, peptide YY, glucagon-like peptide-1, and other appetite-regulating hormones, together with increased hunger and diminished satiety [11,12,13,20,21,22,23]. Importantly, these physiological responses may persist for years after initial weight reduction, creating a biological environment favoring weight regain despite continued behavioral efforts [13]. Individuals achieving greater early weight loss may represent a subgroup with superior adherence to lifestyle recommendations, more favorable behavioral adaptations, greater treatment engagement, or biological characteristics that facilitate sustained weight reduction despite these compensatory responses.

Behavioral factors are equally important in explaining long-term weight trajectories. Successful weight maintenance depends not only on initial dietary adherence but also on sustained engagement in physical activity, self-monitoring, regular clinical follow-up, and long-term behavioral modification [9,10,16,17]. Previous investigations have consistently demonstrated that continued self-monitoring, frequent contact with healthcare professionals, and maintenance of high physical activity levels are associated with improved long-term outcomes [16,17]. Because the present study reflects routine clinical practice rather than a controlled research intervention, variability in adherence to scheduled visits and lifestyle recommendations probably contributed substantially to the observed heterogeneity in weight trajectories. Consequently, early weight loss should not be interpreted as a causal determinant of future outcomes but rather as an integrated clinical marker reflecting the combined influence of biological responsiveness, behavioral adherence, and treatment engagement.

Our findings are also consistent with the contemporary conceptualization of obesity as a chronic, relapsing disease requiring long-term management rather than a condition successfully treated through short-term weight reduction alone [1,2,4,5]. Current international guidelines emphasize that long-term obesity management should include continuous monitoring, individualized behavioral support, timely treatment intensification when clinically indicated, and recognition that weight regain represents an expected component of the natural history of obesity rather than treatment failure [1,2,4]. Within this framework, early weight loss may help clinicians identify individuals requiring closer follow-up, additional behavioral support, or other appropriate treatment-intensification strategies before clinically significant weight regain develops. Where clinically appropriate, treatment intensification may also include evidence-based pharmacological therapies, such as glucagon-like peptide-1 receptor agonist or dual incretin-based treatment, although patients receiving anti-obesity medications were excluded from the present study [32,33].

Interpretation of the present findings should also consider the considerable methodological heterogeneity that characterizes the existing literature on weight regain. Previous studies have used multiple definitions based on absolute weight regain, percentage of baseline weight regained, percentage of maximum weight loss regained, or changes relative to nadir body weight, resulting in limited comparability across investigations [18]. In the present study, clinically significant weight regain was defined using the weight regain ratio, calculated relative to nadir weight and baseline weight, thereby accounting for the magnitude of the initial weight loss. This approach aligns with recommendations advocating greater standardization of obesity outcome reporting and may facilitate more meaningful comparisons across future studies [2,18].

Several limitations should be acknowledged. First, the retrospective observational design precludes causal inference and remains susceptible to residual confounding despite multivariable adjustment. Second, substantial attrition occurred during routine clinical follow-up because the primary analysis required complete longitudinal weight measurements from baseline through one year. Consequently, the final analytical cohort represented only a subset of the original study population and may have been enriched for individuals with greater adherence to treatment and follow-up. Consistent with this possibility, included participants were younger and had a slightly higher baseline body mass index than those excluded, indicating the potential for selection bias and limiting the generalizability of the findings. Third, only nine participants experienced clinically significant weight regain, resulting in limited statistical power and wider confidence intervals and less precise effect estimates. Although sensitivity analyses, including Firth-penalized logistic regression, yielded findings consistent with the primary analysis, the possibility of model instability cannot be completely excluded, and external validation in larger cohorts is warranted. Fourth, detailed information regarding adherence to lifestyle recommendations, objectively measured physical activity, psychosocial characteristics, socioeconomic factors, and body composition was not consistently available in the electronic medical records and therefore could not be incorporated into the regression models. These unmeasured factors may have influenced both early weight loss and subsequent weight regain. Finally, this was a single-center study conducted at a tertiary referral obesity clinic, and the findings should therefore be interpreted as exploratory and hypothesis-generating until confirmed in larger prospective multicenter studies with standardized follow-up and comprehensive assessment of potential confounding factors.

Despite these limitations, the study possesses several important strengths. It was conducted within a specialized multidisciplinary obesity center using standardized clinical assessments under routine healthcare conditions, providing real-world evidence complementary to randomized trials [9,10]. The primary outcome was defined using a clinically meaningful weight regain ratio based on nadir body weight rather than absolute body weight change, reducing potential misclassification of weight regain. The statistical analysis incorporated prespecified multivariable adjustment, sensitivity analyses using an alternative outcome definition, and Firth-penalized logistic regression to evaluate the robustness of the findings. Furthermore, reporting followed the STROBE recommendations for observational studies, improving transparency and reproducibility [29]. Baseline characteristics of included and excluded participants were formally compared to evaluate potential selection bias arising from complete-case analysis.

Future prospective multicenter studies with larger sample sizes, standardized follow-up protocols, and comprehensive assessment of behavioral, metabolic, hormonal, genetic, and pharmacological factors are needed to validate these findings and determine whether incorporating early weight loss into clinical prediction models can improve long-term obesity management and weight maintenance across diverse healthcare settings.

Overall, the present findings support the concept that the initial response to lifestyle-based obesity treatment provides clinically relevant prognostic information regarding subsequent weight maintenance. Although early weight loss should not be viewed as a deterministic predictor of future outcomes, it may represent a simple, inexpensive, and readily available clinical marker capable of identifying patients who could benefit from intensified long-term follow-up and individualized obesity management strategies.

## 5. Conclusions

In this retrospective cohort study conducted in a real-world multidisciplinary obesity care setting, greater early weight loss achieved during the first three months of treatment was independently associated with a lower likelihood of clinically significant weight regain after one year. Because early weight loss is routinely assessed in clinical practice, it may represent a simple, inexpensive, and readily available clinical marker for identifying individuals who could benefit from closer follow-up and individualized obesity management. However, these findings should be interpreted cautiously because of the retrospective design, complete-case analysis, substantial loss to follow-up, and limited number of outcome events. Accordingly, the results should be regarded as exploratory and hypothesis-generating until confirmed in larger prospective multicenter studies with standardized follow-up and comprehensive assessment of behavioral, metabolic, and pharmacological factors.

## Figures and Tables

**Table 1:** Baseline demographic and clinical characteristics of the study participants according to clinically significant weight regain at one year.

Characteristic	No Weight Regain (*n* = 70)	Clinically Significant Weight Regain (*n* = 9)	*p*-Value
Age (years)	38.0 ± 11.0	39.3 ± 10.0	0.745
Female sex, *n* (%)	64 (91.4)	9 (100.0)	1.000
Baseline body weight (kg)	105.3 ± 15.0	96.3 ± 13.4	0.110
Body mass index (kg/m^2^)	41.3 ± 5.4	38.7 ± 4.3	0.156
Early weight loss (%)	7.7 ± 5.5	2.4 ± 2.3	<0.001
Weight regain ratio (WRR)	0.07 ± 0.12	1.02 ± 0.55	0.002

Abbreviations: WRR, weight regain ratio.

**Table 2:** Univariable logistic regression analysis of factors associated with clinically significant weight regain at one year.

Variable	Odds Ratio (OR)	95% CI	*p*-Value
Early weight loss (%)	0.815	0.688–0.965	0.018

Abbreviations: OR, odds ratio; CI, confidence interval.

**Table 3:** Multivariable logistic regression analysis of factors associated with clinically significant weight regain at one year.

Variable	Adjusted OR	95% CI	*p*-Value
Early weight loss (%)	0.784	0.646–0.950	0.013
Age (years)	1.036	0.957–1.120	0.381
Baseline BMI (kg/m^2^)	1.047	0.802–1.368	0.734
Baseline body weight (kg)	0.955	0.869–1.048	0.332

Abbreviations: OR, odds ratio; CI, confidence interval; BMI, body mass index.

**Table 4:** Sensitivity analyses evaluating the robustness of the association between early weight loss and clinically significant weight regain.

Sensitivity Analysis	Adjusted OR	95% CI	*p*-Value
Primary multivariable model (WRR ≥ 0.50)	0.784	0.646–0.950	0.013
Female-only analysis	0.790	0.660–0.940	0.008
Alternative outcome definition (WRR ≥ 0.25)	0.950	0.870–1.050	0.315
Firth-penalized logistic regression	0.814	0.686–0.966	0.019

Abbreviations: OR, odds ratio; CI, confidence interval; WRR, weight regain ratio.

**Table 5:** Firth-penalized logistic regression analysis of factors associated with clinically significant weight regain at one year.

Variable	Adjusted OR	95% CI	*p*-Value
Early weight loss (%)	0.814	0.686–0.966	0.019
Age (years)	1.033	0.954–1.118	0.418
Baseline body mass index (kg/m^2^)	1.041	0.799–1.357	0.771
Baseline body weight (kg)	0.958	0.872–1.052	0.369

Abbreviations: OR, odds ratio; CI, confidence interval.

## Data Availability

The datasets generated and/or analyzed during the current study are not publicly available because they contain potentially identifiable clinical information. De-identified data are available from the corresponding author upon reasonable request and with permission from the institutional ethics committee.

## Supplementary Materials

The following supporting information can be downloaded at: https://www.mdpi.com/article/10.3390/medicina62081450/s1, Table S1. Comparison of baseline characteristics between participants included in the final analytical cohort and those excluded because of incomplete longitudinal follow-up.

## Abbreviations

BMI	Body Mass Index
CI	Confidence Interval
HbA1c	Glycated Hemoglobin
IQR	Interquartile Range
OR	Odds Ratio
ROC	Receiver Operating Characteristic
SD	Standard Deviation
STROBE	Strengthening the Reporting of Observational Studies in Epidemiology
WRR	Weight Regain Ratio
WHO	World Health Organization
