# Supplementary material for: Association Between Early Weight Loss and One-Year Weight Regain After Lifestyle-Based Obesity Treatment: A Retrospective Cohort Study
Source: Medicina (Kaunas). 2026 Jul 26;62(8):1450. doi: 10.3390/medicina62081450 (PMC13514555; doi:10.3390/medicina62081450)
Supplement: Supplementary file 1 [file medicina-62-01450-s001.zip › medicina-4440183-supplementary.pdf]

Supplementary Table S1

Title: Comparison of baseline characteristics between participants included in the final analytical cohort and those excluded because of incomplete longitudinal follow-up.

Characteristic	Included (n = 79)	Excluded (n = 952)	p-value
Age (years), mean ± SD	38.1 ± 10.9	41.0 ± 11.7	0.025
Female sex, n (%)	76 (96.2)	846 (88.9)	0.065
Baseline body weight (kg), mean ± SD	104.3 ± 15.1	102.4 ± 17.3	0.299
Baseline BMI (kg/m^2), mean ± SD	41.0 ± 5.3	38.9 ± 6.0	0.002

Abbreviations: BMI, body mass index; SD, standard deviation.

Statistical methods: Continuous variables were compared using the independent-samples t-test. Categorical variables were compared using Pearson's chi-square test or Fisher's exact test, as appropriate.

Legend: Baseline characteristics of participants included in the final analytical cohort were compared with those of patients excluded because of incomplete longitudinal follow-up. Included participants were significantly younger and had a slightly higher baseline body mass index, whereas baseline body weight and sex distribution did not differ significantly between groups. These findings suggest that the analytical cohort may represent patients with greater adherence to routine clinical follow-up and should therefore be interpreted in the context of potential selection bias.
